# Institutional priorities and student engagement: a multi-stakeholder analysis of physical education in Israel

**DOI:** 10.3389/fspor.2025.1625231

**Published:** 2025-08-01

**Authors:** Ali Alayan, Ahmad Salhab, Paz Carmel, Ronit Ahdut HaCohen

**Affiliations:** ^1^ Department of Education, Adam Mickiewicz University, Poznan, Poland; ^2^ Department of Science, David Yellin Academic College of Education, Jerusalem, Israel; ^3^ Department of Adult Education, David Yellin College of Education, Jerusalem, Israel

**Keywords:** physical education, student perceptions, school-based physical activity, stakeholder attitudes, Israeli education system

## Abstract

**Background:**

Physical Education (PE) is crucial to the development of children and adolescents, promoting physical health, mental well-being, and academic achievement. Despite global guidelines advocating for structured physical activity (PA) in schools, PE often remains underprioritized, especially in systems with competing academic demands. This study investigates the perceptions of students, PE teachers, and school administrators in Israel regarding PE value, participation, and institutional support.

**Methods:**

A cross-sectional survey was conducted with 150 participants from six East Jerusalem secondary schools, including students (70%), PE teachers (20%), and administrators or other staff (10%). A validated questionnaire assessed attitudes toward PE, including actual and preferred instructional hours, extracurricular engagement, and perceived barriers. Instrument reliability was confirmed via Cronbach's alpha coefficients (≥0.70), and data were analyzed using descriptive statistics and chi-square tests.

**Results:**

Although 83% of students rated PE as equally important as other academic subjects, 70.7% reported receiving only one hour per week. Students with more PE exposure were significantly more likely to engage in extracurricular PA, value PA, and feel that they have more accessible and supportive opportunities for sports participation in their surroundings (*p* < 0.01). Cross-group comparisons revealed perceptual gaps in students who reported lower environmental support than teachers and administrators (*p* = 0.003). While PE teachers were seen as encouraging in class, their influence outside scheduled hours was less evident. Students noted institutional barriers, including frequent cancellations of PE classes and limited extracurricular support. Nonetheless, enjoyment of PE remained high, with most barriers viewed as having only a minor impact.

**Conclusion:**

These findings highlight the need for expanded PE hours, more substantial institutional commitment, and coordinated stakeholder action to foster an active school culture in Israel. However, limitations such as the modest sample size and the geographic concentration in East Jerusalem may affect the generalizability of the findings.

## Introduction

Physical Education (PE) is a cornerstone of children's overall development, contributing significantly to physical health, mental well-being, and social growth. Despite its recognized benefits, PE remains underemphasized in formal education settings worldwide (1–10). Structured PE classes not only support the physical competencies of students but also facilitate adherence to exercise guidelines recommended by international health organizations (11). Moreover, consistent engagement in physical activity (PA) has been associated with a reduced risk of chronic conditions such as obesity, diabetes, and cardiovascular disease and is linked to improved cognitive function, psychological health, and academic performance (12–15). Nonetheless, PE continues to be viewed as one of the least prioritized subjects in schools globally (16). Many researchers have noted a drop in the active participation of children and adolescents in physical activities due to sedentary behaviors, increased screen time, and minimal opportunity for fun activities (17–19). Compared with other subjects, PE receives less curriculum time in primary and secondary schools and is constrained by limited financial resources, policy noncompliance, and competing curricular demands (20, 21). Furthermore, disparities in access to facilities, teaching materials, qualified professionals, and community support pose additional challenges to delivering effective PE, particularly in underserved or marginalized regions (22, 23). Despite these logistical barriers and the profound benefits PE offers for physical, mental, and social well-being, it has long occupied a marginalized position within global education systems (23, 24). Often undervalued and mischaracterized as recreational rather than academic, PE is frequently allocated fewer resources, instructional hours, and institutional recognition than other core disciplines (24). The COVID-19 pandemic, however, highlighted the urgent need for structured PA and reaffirmed PE's essential role in public health (24). Lockdowns triggered significant declines in PA levels among youth, contributing to widespread physical and mental health deterioration, and prompting global health organizations such as WHO and UNESCO to call for renewed investment in quality PE (22–24). As Shirotriya and Beighle emphasize, this period presents a pivotal opportunity to reframe PE not as a peripheral subject, but as a central pillar in promoting lifelong health and educational equity (24).

In Israel, the delivery of consistent and high-quality PE programs is hindered by a combination of systemic, cultural, and logistical barriers (25, 26). Systemic issues include unequal resource allocation across different educational sectors, particularly between Jewish and Arab schools, as well as variability in curriculum enforcement. Cultural attitudes toward PE often deprioritize PA in favor of academic subjects, especially in communities where PE is not viewed as essential to student success. Logistical constraints further complicate implementation, including overcrowded classrooms, limited access to gymnasiums or open spaces, scheduling conflicts, and inadequate transportation for inter-school activities. These challenges are particularly evident in diverse regions such as East Jerusalem, where social, cultural, and political complexities intersect to limit institutional support for PE. Despite the Ministry of Education's requirement for PE classes, substantial variation remains in the frequency and quality of PE instruction across schools, educational sectors, and grade levels (25, 26).

These variations, coupled with minimal research on how students, teachers, and administrators perceive the role and importance of PE, create a significant knowledge gap. Understanding these stakeholder perspectives is crucial for identifying the barriers to consistent policy implementation and for informing interventions that promote a robust and active school culture. Some schools allocate no more than one to two hours a week for physical activities, reflecting the divergence between policy and practice (27, 28). Communities, school-based policies, and teacher training are vital in encouraging routine PA participation (29–31). Nonetheless, there is scant evidence on how different educational system stakeholders - students, PE teachers, and school principals - perceive the importance and contribution of PE classes to education in Israel. Understanding these stakeholders and their perceptions is equally essential, as the sentiments of these stakeholders strongly influence the attention, resources, quality, and efficacy accorded to PE programs. Regarding attendance and participation in PE activities, students are influenced by several factors, including personal and social aspects such as having a positive self-concept, peer support, and the extent to which the school promotes physical activities (32). Job satisfaction among PE teachers is closely tied to their professional environment, including administrative support, availability of resources, and opportunities for professional development (33, 34). Research indicates that supportive leadership, equitable time allocation for PE, and inclusion in decision-making processes significantly influence teachers' motivation and retention (34). Moreover, perceived status and institutional recognition of PE as a legitimate academic subject directly impact teachers' sense of value and commitment (33). As principal decision-makers, school administrators play a pivotal role in shaping this environment by determining PE's curricular priority, funding, staffing, and policy implementation (34). A lack of administrative support or prioritization often results in diminished morale and limited instructional effectiveness.

This study aims to explore and evaluate the perspectives of key stakeholders on institutional priorities and student engagement in PE across the Israeli school system. Using a structured questionnaire, we assess students' views on PE, the number of hours they attend and wish to attend, their extracurricular PA, and their perceptions of school and teacher support. The study also captured the perspectives of PE teachers and school administrators, providing an institutional account of priorities, barriers, and strategies to enhance participation. Through this multi-stakeholder lens, the study seeks to address gaps in policy and practice, offer evidence-based recommendations, and support the development of more inclusive and effective PE programs in Israel. Although the research focused primarily on secondary schools in East Jerusalem, the findings offer actionable insights that can guide differentiated implementation strategies across educational sectors and age groups by identifying specific challenges, contextual needs, and stakeholder attitudes that may vary between Arab and Jewish sectors or between younger and older students.

## Methods

### Study population

A cross-sectional survey was conducted among 150 respondents from six East Jerusalem secondary schools that follow the Israeli curriculum (Table 1). The largest subgroup consisted of students (*n* = 105, 70%), followed by PE teachers (*n* = 30, 20%). School administrators formed 4% (*n* = 6) of the sample. Additionally, 6% (*n* = 9) were categorized as other stakeholders, referring to teachers from non-PE subjects who nonetheless provided essential insights into students' interdisciplinary perceptions of PE.

**Table 1:** Distribution of study participants by stakeholder role.

Category	Frequency	Percentages (%)
PE teacher	30	20
Student	105	70
School administrator	6	4
Other stakeholders	9	6

### Sampling strategy and sample size determination

A multi-stage random sampling approach was employed to recruit participants exclusively from secondary schools in East Jerusalem, which are administratively under Israeli jurisdiction. These schools typically prepare students for the Israeli Bagrut (matriculation) examinations. To receive a grade in PE, students are generally required to participate in weekly PE classes, with assessment based on participation and performance in fitness or skill-based activities, subject to school-level guidelines. The local education authority obtained an official list of all 10 secondary schools in East Jerusalem. Six schools were selected: Beit Hanina, Shuafat, Wadi al-Joz, Sur Baher, Silwan, and Jabal Mukaber. Within each selected school, participants were randomly drawn from four stakeholder categories: students, PE teachers, administrators, and other staff, with a target of at least 25 participants per school.

Prior to data collection, the required sample size was estimated using G*Power (Version 3.1.9.7), assuming a moderate effect size (Cohen's w = 0.3), *α* = 0.05, and power = 0.80. This calculation indicated a minimum sample of 138 participants; therefore, the final target was 150 to account for potential non-responses and incomplete data. PE classes in these schools typically include a range of structured physical activities designed to promote cardiovascular endurance, muscular strength, flexibility, and coordination. Typical activities include running, stretching, push-ups, sit-ups, pull-ups, and team sports such as soccer, basketball, and volleyball.

### Questionnaire development and validation

The questionnaire was developed by the research team based on a comprehensive review of relevant literature on school-based PA and PE. Items were adapted from previously validated international and Israeli instruments to ensure cultural relevance to the East Jerusalem school context. To assess content validity, the instrument was reviewed by a panel of 3 experts in PE, educational psychology, and curriculum design, all with more than five years of academic or field experience. They evaluated each item for clarity, relevance, and alignment with the study's objectives. Based on their feedback, several items were revised or removed to enhance precision and minimize ambiguity.

To ensure face validity, the revised questionnaire was pilot-tested with a convenience sample of 15 students from non-participating schools in East Jerusalem. Students were asked to comment on the clarity, language, and interpretability of the items. Minor modifications were made based on their suggestions. The questionnaire demonstrated acceptable reliability, with a Cronbach's alpha of 0.87.

### Questionnaire reliability

Cronbach's alpha coefficients were calculated for each of the four primary domains assessed in the questionnaire to guarantee the credibility and internal consistency of the study instrument (Table 2).

**Table 2:** Internal consistency (cronbach's alpha) for the questionnaire fields.

Fields	Cronbach's alpha test	Number of items	Sample size
Students total PA	0.763	4	150
Perceptions about the importance of PA	0.711	4	150
Activities to encourage students to participate in PA	0.849	6	150
Perceptual factors that affect students’ participation in education classes	0.727	12	150

The domain “Activities to encourage students to participate in PA” demonstrated excellent internal consistency, with a Cronbach's alpha of *α* = 0.849. The “Students’ total PA” domain showed good reliability (*α* = 0.763). Additionally, the domains “Perceptions about the importance of PA” and “Factors influencing students’ participation in PE classes” reported acceptable reliability coefficients of *α* = 0.711 and *α* = 0.727, respectively. All coefficients exceeded the widely accepted threshold of 0.70, indicating that the items within each construct were internally consistent and yielded stable measurements. These findings support the instrument's psychometric robustness and its suitability for assessing perceptions and behaviours related to PE.

### Ethical considerations

This study received ethical approval from the Chief Scientist's Office at the Israeli Ministry of Education, the official body responsible for authorizing research conducted within the national school system. As part of this approval process, the research team received formal authorization to enter schools, which was presented to school administrators to introduce the study and obtain institutional support. Informed consent was obtained from the parents or legal guardians of all participating students. An official letter, distributed to families, clearly outlined the study's objectives, procedures, and confidentiality measures and included an opt-out form. Students whose parents declined participation were excluded from the study, in full compliance with the approved ethical protocol. All research activities were conducted by qualified academic investigators. Focus group discussions, where applicable, were facilitated by trained moderators approved under the ethics clearance to ensure methodological and ethical integrity throughout the study.

### Statistical analysis

Descriptive statistics (means, standard deviations, and frequencies) were used to characterize respondents' demographics and attitudes. Chi-square tests were conducted to assess associations among categorical variables. All analyses were performed using SPSS version 26, with statistical significance set at *p* < 0.05.

## Results

### Student valuation of PE as a key driver of engagement: beyond exposure and environment

As indicated in Table 3, in terms of PE hours per week, a significant majority of the sample population (*n* = 106, 70.7%) noted having only one hour, whereas two hours (*n* = 22, 14.7%) or three or more hours (*n* = 22, 14.7%) were lesser reported. These numbers suggest that almost three out of four students have low PA levels within the school system. In terms of preferences, most participants (*n* = 98, 65.3%) were satisfied with the current distribution of hours. Almost one-third of the students (*n* = 47, 31.3%) wanted more PE hours, while only a small number (*n* = 5, 3.3%) would rather have fewer. This highlights a slight disparity between actual provision and students' perceived need for PE, which is generally beneficial for students' attitudes towards exercise within schools. To gain further insight into the students' views, they were asked to assess the value associated with PE and their level of participation in physical activities (Table 4). The statement “PE classes are important to you and are no less important than any other subject taught in school” received the highest mean score (M = 4.17, SD = 0.91; relative weight = 83%). It was followed by the statement, “Compared to other subjects, PE classes are just as important as all other disciplines” (M = 3.54, SD = 1.00; relative weight = 71%). Strong agreement was noted regarding the importance of PE. However, the participation outside the school hours was quite different. The statement “PA in the after-work hours…” had the lowest mean score (M = 1.69, SD = 0.84; relative weight = 56%), and “Sports activities in which students can engage in their social surroundings” also had low mean scores (M = 2.93, SD = 1.13). The average score (M = 3.36, SD = 0.57) indicates that the perception of PE is generally positive, although additional factors make participation outside of set school hours challenging.

**Table 3:** Distribution of participants according to the actual and preferred number of PE hours per week.

Variables	Category	Frequency	Percentages (%)
PE hours	One hour	106	70.7
	Two hours	22	14.7
	Three hours or more	22	14.7
"I would have liked the number of hours of…	More or much more than it is today	47	31.3
	The same thing without any differences	98	65.3
	Less or much less than it is today	5	3.3

**Table 4:** Descriptive statistics of participants’ perceptions regarding PE and PA.

Answer	Mean ± SD	Relative weight (%)	Order	Degree
Sport activities which students can participate in their surrounded environment	2.9267 ± 1.13	59%	3	With equal percent
PA in the after-work hours as part of an extracurricular activity or in a free manner	1.6867 ± 0.84	56%	4	I participate in any PA (sometimes)/or free activity (once a week).
PE classes are important to you and are no less important than any other subject taught in school	4.1667 ± 0.91	83%	1	Agree
Compared to other subjects, PE classes are just as important as all other disciplines	3.5400 ± 1	71%	2	With large percent

To understand how these perceptions vary across roles, a cross-tabulation analysis was conducted among PE teachers, students, and administrators (Table 5). Statistically significant differences emerged regarding the perceived availability of community sports resources: while 79.6% of students reported “very little/little” access, only 3.7% of PE teachers and 19.7% of administrators held the same view (χ^2^ = 16.375, *p* = 0.003). Teachers were more likely to report moderate or high availability. A similar perceptual gap was observed in engagement with after-school activities. Although 64.3% of students reported no participation, PE teachers indicated more diverse activity patterns. While not statistically significant, the difference approached relevance (χ^2^ = 8.765, *p* = 0.067). Agreement on the value of after-school PA also varied by role (χ^2^ = 8.839, *p* = 0.065), with students expressing higher levels of enthusiasm than teachers or administrators. Finally, significant differences were observed in the perceived academic importance of PE (χ^2^ = 16.823, *p* = 0.002), with teachers more likely to rate it as “large” or “very large”, whereas administrators and students were somewhat more varied.

**Table 5:** Cross-tabulation of stakeholder responses by role (PE teachers, students, school administrators/others) and Chi-square test results.

Item		Stakeholder role						Chi-square	*P*-value
		PE teacher		Student		School administer/or other			
		freq	%	freq	%	freq	%		
Sport activities which students can participate in their surrounded environment	Very little/little percent	2	3.7	43	79.6	9	19.7	16.375	0.003
	Equal percentage	17	30.4	35	62.5	4	7.1		
	Large/very large percent	11	27.5	27	67.5	2	5		
PA in the after-work hours as part of an extracurricular activity or in a free manner* State1 Cross tabulation	I did not participate	20	23.8	54	64.3	10	11.9	8.765	0.067
	Once a week	7	24.1	18	62.1	4	13.8		
	Two or three times a week	3	8.1	33	89.2	1	2.7		
PA in the after-work hours as part of an extracurricular activity or in a free manner	Disagree	2	33.3	4	66.7	0	0	8.839	0.065
	Neutral	0	0	16	100	0	0		
	Agree	28	21.9	85	66.4	15	11.7		
Compared to other subjects, PE classes are just as important as all other disciplines	Very little/little percent	3	13	20	87	0	0	16.8	0.002
	Equal percentage	2	5	35	87.5	3	7.5		
	Large/very large percent	30	20	105	70	15	10		

To assess whether exposure to PE shapes students' perceptions and behaviours, another cross-tabulation examined outcomes by the number of weekly PE hours (Table 6). Students receiving three or more PE hours were significantly more likely to report high availability of sports opportunities in their surrounding environment (32.5%) compared to those with two hours (15%) and one hour (11.1%) (χ^2^ = 15.158, *p* = 0.004). In contrast, 74.1% of students with only one PE hour reported limited access to such opportunities. Although students with greater PE exposure also reported higher rates of participation in extracurricular PA two to three times per week (14.5% for the three-hour group), the association did not reach statistical significance (χ^2^ = 8.938, *p* = 0.177). However, perceptions regarding the value of after-school PA differed significantly across exposure levels (χ^2^ = 300, *p* < 0.0001). All students who disagreed with the value of after-school activity were from the one-hour group, whereas agreement was exclusive to students with two or more hours of PE. Likewise, perceived academic importance of PE was strongly associated with exposure (χ^2^ = 56.393, *p* < 0.0001), with 46.4% of students in the three-hour group and 37.7% in the two-hour group rating it as “large” or “very large”, compared to only 15.9% in the one-hour group. These findings indicate that increased PE exposure is significantly linked to more favorable attitudes toward both PA and the academic value of PE. To deepen the analysis, students' preferences regarding weekly PE hours were examined in relation to their PA attitudes and behaviors (Table 7). Preferences were categorized as wanting more, the same, or fewer PE hours. A statistically significant association was observed between students’ attitudes toward after-school activity and their preferences (χ^2^ = 26.680, *p* < 0.001). Among those who valued after-school activities, 31.3% wanted more PE, and only 0.8% wanted less. In contrast, 33.3% of students who did not value after-school activities preferred fewer hours. A similar trend was observed regarding how students perceived the academic importance of PE (χ^2^ = 20.210, *p* < 0.001). Students who rated PE as “very important” overwhelmingly wanted either the same amount (65.3%) or more time (31.3%), while only 3.3% preferred less. Meanwhile, 17.4% of those rated PE as “very little/little” in importance preferred reduced hours. Other variables, such as the perceived availability of sports opportunities and actual participation in extracurricular PA, did not show statistically significant associations with students' preferences for PE hours. This suggests that while environmental factors may shape attitudes, students' valuation of PE and belief in its importance are the strongest predictors of their desire for increased PE time.

**Table 6:** Cross-tabulation of participants’ responses by the number of PE hours per week.

Item		Number of hours of PE at the school						Chi-square	*P*-value
		One hour		Two hours		Three or more			
		freq	%	freq	%	freq	%		
Sport activities which students can participate in their surrounded environment	VERY little/little percent	40	74.1	8	14.8	6	11.1	15.158	0.004
	EQUAL percentage	45	80.4	8	14.3	6	5.3		
	LARGE/very large percent	21	52.5	6	15	13	32.5		
PA in the after-work hours as part of an extracurricular activity or in a free manner *State1 Crosstabulation	I did not participate	6	100	0	0	0	0	8.938	0.177
	Once a week	15	93.8	1	6.3	0	0		
	Two or three times a week	48	69.6	11	15.9	10	14.5		
PA in the after-work hours as part of an extracurricular activity or in a free manner	Disagree	6	100	0	0	0	0	300	<0.0001
	Neutral	0	0	16	100	0	0		
	Agree	0	0	69	100	0	0		
Compared to other subjects, PE classes are just as important as all other disciplines	very little/little percent	2	33.3	2	33.3	2	33.3	56.393	<0.0001
	equal percentage	8	50	8	50	0	0		
	Large/very large percent	11	15.9	26	37.7	32	46.4		

**Table 7:** Cross-tabulation of students’ preferences for the number of weekly PE hours with their PA perceptions and behaviors.

Item		How the student like for the number of hours of PE						Ch-square	*P*-value
		More		The same		Less			
		freq	%	freq	%	freq	%		
In the area where I live, there is a wide choice of sports activities that students can participate in	Very little/little percent	17	31.5	34	63	3	5.6	4.045	0.400
	Equal percentage	20	35.7	36	64.3	0	0		
	Large/very large percent	10	25	28	70	2	5		
PA in the after-work hours as part of an extracurricular activity or in a free manner * State1 Crosstabulation	I did not participate	27	32.1	54	64.3	3	3.6	4.211	0.378
	Once a week	6	20.7	21	72.4	2	6.9		
	Two or three times a week	14	37.8	23	62.2	0	0		
PA in the after-work hours as part of an extracurricular activity or in a free manner	Disagree	0	0	4	66.7	2	33.3	26.68	0.000
	Neutral	7	43.8	7	43.8	2	12.5		
	Agree	40	31.3	87	68	1	0.8		
Compared to other subjects, PE classes are just as important as all other disciplines	Very little/little percent	9	39.1	10	43.5	4	17.4	20.21	0.000
	Equal percentage	15	37.5	25	62.5	1	1.1		
	Large/very large percent	47	31.3	98	65.3	5	3.3		

### Schools motivate students more during class time than after hours

The previous analyses examined the differences stemming from stakeholder roles in the PE classes, the number of hours spent in class, and whether students would prefer more or fewer PE classes. Now, it is essential to examine students' attitudes towards the active role the school plays in encouraging them to engage in PA. These attitudes reflect the school's institutional commitment to student physical well-being. To understand this aspect, the focus was on the descriptive statistics regarding the level to which schools motivate, instruct, and facilitate PA both during and after school hours. A descriptive analysis was made to assess how students working in the school perceive the encouragement given towards exercise both within and outside official school hours (Table 8). The item rated highest was “During the school work plan, the stresses motivating students to participate in sports activity inside and outside the school”, which had a mean value of 3.78 (SD = 0.99), with 75.6% agreement, indicating a high level of agreement. In the same context, students have also confirmed that “The school explains the importance of PA and a healthy lifestyle” with a mean score of 3.67 (SD = 1.07; relative weight = 73.5%), which suggests moderate agreement. There was moderate agreement as well for the item “Your school encourages students to play ball games or free games in the schoolyard” (M = 3.47, SD = 1.21) but the lowest mean was for “Your school encourages students to participate in PA also after official working hours”, which received a neutral response (M = 3.13, SD = 1.19; relative weight = 63%). Based on these results, students perceive that motivation and structured education about PA are mainly limited to school hours, and there is little to no direct encouragement to participate in physical activities after school. The overall average is somewhat high, with a mean item score of 3.51 (SD = 0.82), indicating that there is general agreement that schools provide PE; however, the level of such encouragement varies depending on the situation.

**Table 8:** Descriptive statistics of students’ perceptions of school-based encouragement for PA.

The answer	Mean	Standard deviation	Relative weight	Order	Degree
During the school work plan, the stresses motivating students to participate in sports activity inside and outside the school	3.7800	0.99576	75.6	1	Agree
The school will explain to students the importance of PA and a healthy lifestyle	3.6733	1.07126	73.5	2	Agree
Your school encourages students to play ball games or free games in the schoolyard	3.4667	1.20773	69	3	Agree
Your school encourages students to participate in PA also after official working hours, for example, extracurricular activities	3.1267	1.19449	63	4	Neutral

To deepen this analysis, a cross-tabulation was conducted to examine differences among students, PE teachers, and school administrators. Statistically significant group differences were observed across all four items (Table 9). Teachers and students expressed relatively higher agreement with items related to in-school encouragement, while administrators showed notably lower deal. For example, although 61.7% of students agreed that PA is emphasized within the school plan, only 10.3% of administrators concurred (χ^2^ = 21.681, *p* = 0.000). Similarly, students and teachers were more likely to agree that the school promotes ball games or explains the importance of a healthy lifestyle, whereas administrators were less likely to affirm these roles. The starkest perception gap appeared in encouragement for after-hours activity, where 82% of students disagreed that schools promoted such engagement, despite 30.6% of teachers reporting otherwise (χ^2^ = 17.819, *p* = .001).

**Table 9:** Cross tabulation for the question: stakeholder role vs. Perceptions about the importance of PA.

Paragraph		Situation						Ch-square	Probability value
		PE teacher		Student		School administer/or other			
		freq	%	freq	%	freq	%		
During the school work plan, he stresses on motivating students to participate in sports activity inside and outside the school	Disagree	0	0	14	77	4	22.2	21.681	.000
	Neutral	0	0	25	100	0	0		
	Agree	30	28	66	61.7	11	10.3		
The school will explain to students the importance of PA and a healthy lifestyle	Disagree	0	0	24	100	0	0	27.266	.000
	Neutral	1	4	24	96	0	0		
	Agree	29	28.7	57	56.4	15	14.9		
Your school encourages students to play ball games or free games in the schoolyard	Disagree	6	17.1	27	77.1	2	5.7	12.136	.016
	Neutral	1	4.3	22	95.7	0	0		
	Agree	23	25	56	60.9	13	14.1		
Your school encourages students to participate in PA also after official working hours, for example extracurricular activities	Disagree	5	10	41	82	4	8	17.819	.001
	Neutral	3	10.7	25	89.3	0	0		
	Agree	22	30.6	39	54.2	11	15.3		

Taken together, the descriptive and inferential analyses reveal that while students acknowledge the presence of PA promotion during school hours, the effort appears to taper off outside these times. Additionally, significant perceptual mismatches between stakeholder groups underscore the need for greater alignment in communication and policy regarding PA promotion across the school day.

### Students view PE teachers as motivators within the class, but are less engaged

To assess how students perceive the role of PE teachers in encouraging PA beyond scheduled class periods, a series of items was analysed using descriptive statistics (Table 10). The analysis focused on teacher encouragement, educational messaging, follow-up on extracurricular engagement, and the frequency of active breaks during the school day. The highest-rated item was “The PE teacher at school encourages me to do PA in my spare time”, with a mean score of 3.47 (SD = 1.12) and a relative weight of 0.694. This suggests that students agree that their PE teachers serve as motivational figures for promoting PA outside of school hours. The second-highest rated statement was “The PE teacher explains the importance of PA” (M = 3.40, SD = 1.17; relative weight = 0.68), indicating a generally positive perception of the teachers' educational role in health promotion. Conversely, students expressed more neutral views on items that required ongoing engagement or the application of PA to everyday life. The item “During the PE classes, they explain to us how to apply PA also outside working hours (e.g., walking instead of riding a car, using stairs instead of the elevator)” received a mean score of 3.25 (SD = 1.26), while “The PE teacher cares and asks about the sports activities that I participate in after official working hours” received a nearly identical mean of 3.24 (SD = 1.32). Both items reflect a lack of strong consensus, suggesting that while students perceive some support, it may be limited in follow-through or practicality. Finally, the statement addressing the frequency of adequate breaks involving PA within the school day received a mean of 3.25 (SD = 1.11), interpreted as approximately “one or two times a week”, which further suggests room for improvement in incorporating PA into the daily school routine. The overall mean score for this set of items was 3.31 (SD = 0.99), corresponding to a neutral level of agreement. This result indicates that while PE teachers are generally viewed as encouraging, their role in reinforcing PA behaviours beyond class hours may not be fully optimized or consistently experienced by students.

**Table 10:** Descriptive statistics of students’ perceptions regarding the PE teacher's role in promoting PA beyond scheduled class periods.

The answer	Mean	Standard deviation	Relative weight	Order	Degree
The PE teacher in the school explains to us about the importance of PA	3.40	1.17	0.68	2	Agree
The PE teacher at school encourages me to do PA in my spare time	3.47	1.12	0.694	1	Agree
During the PE classes, they explain to us how to apply PA also outside working hours (for example, walking instead of riding the car, climbing stairs instead of using the elevator	3.25	1.26	0.65	4	Neutral
The PE teacher cares and asks about the sports activities that I participate in after official working hours	3.24	1.32	0.648	5	Neutral
How many times a week is an effective break held inside the school?	3.25	1.11	0.65	3	One or two times a week
Overall mean	3.31	0.99	0.662		Neutral

### High enjoyment, low barriers: student perspectives on participation in PE

Although previous studies have analyzed the role schools and PE teachers play in encouraging active lifestyles, students' views on their participation in PE classes and potential restraints are also crucial. Knowing students' perceptions and barriers tells much more about the environment and describes the negative conditions of motivation and participation. The next part of the table examines students' perceptions of their enjoyment of PE and the social, emotional, and structural factors that impact their continued participation. From the analysis of students' responses (Tables 11, 12), it was clear that their overall attitude towards participating in PE was positive, and their perception of barriers to participation was low. Most students supported the statement, “I love participating in PE classes inside the school” (M = 4.15, SD = 0.90; relative weight = 0.83), with this level of support indicating strong enthusiasm for PE within the student body. However, most of the perceived obstacles, such as low motivation (M = 2.26) and insufficient equipment (M = 1.94), as well as teacher training frustration (M = 2.13), and teachers not having time due to numerous other responsibilities (M = 2.07), were rated as having little impact. Likewise, students' low motivation (M = 2.36) or low perceived importance of PE among peers (M = 2.34) were equally as unproblematic. Notably, while the barriers to participation were perceived to be low, one particular concern was the high rate of cancellations of PE compared to other subjects. Students endorsed the statement, “PE classes are canceled more often than other classes” (M = 3.65, SD = 1.28; relative weight = 0.73), suggesting that cancellation of classes may contribute to disaffection. The embarrassment factor in PE classes received a neutral rating (M = 2.91), indicating that respondents perceived emotional discomfort as present, but not to a strong enough extent to influence their participation choice. The overall mean across all items is 2.38 (SD = 0.54), indicating that students generally seem to enjoy PE. Yet, some broader structural or sociological aspects are identifiable and do not allow their full engagement.

**Table 11:** Descriptive statistics of students’ perceptions regarding barriers to participation and attitudes toward PE.

The answer	Mean	Standard deviation	Relative weight	Degree
I love participating in PE classes inside the school	4.15	0.90031	0.83	Agree
Is there a lack of sports infrastructure?	2.26	1.18365	0.57	effect slightly
Is there a shortage of suitable sports equipment?	1.94	.76064	0.49	Effect slightly
There is a shortage of professional teachers in the field of PE	2.18	1.10721	0.55	Effect slightly
Lack of desire among PE teachers for learning and professional development	2.13	1.06010	0.53	Effect slightly
Multiple additional functions performed by a PE teacher	2.07	1.04160	0.52	Effect slightly
Perception of a topic of low importance in the eyes of students	2.34	1.09531	0.587	Effect slightly
Students’ motivation is low when they participate in PE classes	2.36	1.16422	0.59	Effect slightly
In your school, PE classes are repeatedly cancelled compared to other classes, and does the issue affect, in general, the participation of students in PE classes	2.39	1.19616	0.60	Effect slightly
PE classes are cancelled more often than other classes	3.65	1.28485	0.73	Agree
Is there a problem with the lack of encouragement for parents to take their children into PE classes at school?	2.16	0.97011	0.54	Effect slightly
I feel embarrassed in PE classes?	2.91	1.40413	0.582	Neutral
Overall mean	2.38	0.53758		Effect slightly

**Table 12:** Cross-tabulation of stakeholder role and perceptions regarding barriers and attitudes toward PE in schools.

Paragraph		Stakeholder role						Chi-square	*P*-value
		PE teacher		Student		School administer/or other			
		freq	%	freq	%	freq	%		
I love participating in PE classes inside the school	Disagree	0	0	7	100	0	0	8.760	0.067
	Neutral	4	14.8	23	85.2	0	0		
	Agree	26	22.4	75	64.7	15	12.9		
Is there a lack of sports infrastructure?	Slightly or less	13	16.3	59	73.8	8	10	3.380	0.187
	Moderately or more	17	28.3	36	60	7	11.7		
Is there a shortage of suitable sports equipment?	Slightly or less	25	24	66	63.5	13	12.5	7.766	0.021
	Moderately or more	5	13.9	31	86.1	0	0		
There is a shortage of professional teachers in the field of PE	Slightly or less	17	19.5	64	73.6	6	6.9	3.532	0.171
	Moderately or more	13	22.8	35	61.4	9	15.8		
Lack of desire among PE teachers for learning and professional development	Slightly or less	26	30.2	50	58.1	10	11.6	12.095	0.005
	Moderately or more	4	7.1	47	83.9	5	8.9		
Multiple additional functions performed by a PE teacher	Slightly or less	19	21.6	61	69.3	8	9.1	.644	0.725
	Moderately or more	10	17.2	41	70.7	7	12.1		
Perception of a topic of low importance in the eyes of students	Slightly or less	20	24.7	53	65.4	8	9.9	1.792	0.408
	Moderately or more	10	15.6	47	73.4	7	10.9		
Students’ motivation is low when they participate in PE classes	Slightly or less	17	20.7	57	69.5	8	9.8	0.46	0.677
	Moderately or more	13	20	45	69.2	7	10.8		
In your school, PE classes are repeatedly cancelled compared to other classes, and does the issue affect, in general, the participation of students in PE classes	Slightly or less	20	27.4	46	63	7	9.6	4.374	0.112
	Moderately or more	10	13.5	56	75.7	8	10.8		
PE classes are cancelled more often than other classes	Disagree	12	36.4	20	60.6	1	3	10.976	0.029
	Neutral	7	24.1	20	69	2	6.9		
	Agree	11	12.5	65	73.9	12	13.6		
Is there a problem with the lack of encouragement for parents to take their children into PE classes at school?	Slightly or less	20	24.7	49	60.5	12	14.8	7.437	0.024
	Moderately or more	10	15.2	53	80.3	3	4.5		
I feel embarrassed in PE classes	Disagree	14	20.3	47	68.1	8	11.6	3.216	0.522
	Neutral	3	10.3	23	79.3	3	10.3		
	Agree	13	26.5	32	65.3	4	8.2		

## Discussion

This study sought to assess students' perceptions of PE among students, PE teachers, and school administrators in East Jerusalem schools. The findings present a nuanced view of how the educational system values, supports, and implements PE. While there is broad agreement on the importance of PE, key discrepancies emerged between stakeholder roles regarding participation levels, institutional support, and perceived barriers, suggesting a need for more cohesive strategies to promote PA within and beyond the school setting.

A central finding of this study is that the vast majority of students (70.7%) receive only one hour of PE per week, which falls significantly below international recommendations. The World Health Organization (WHO) recommends that children and adolescents engage in at least 60 min of moderate-to-vigorous PA daily (35, 36). According to OECD data, most member countries allocate between 90 and 150 min of PE per week in primary and secondary education (37). For example, countries like France, the UK, and Finland typically provide 2–3 h of PE per week. In contrast, data from the Middle East and North Africa (MENA) region show significant variability, with some countries, such as Qatar and the UAE, implementing two PE lessons per week, while others report less consistent or under-enforced guidelines (38–42). In this context, the limited PE time in East Jerusalem schools raises concerns about whether students receive adequate, structured opportunities to meet health and fitness standards, particularly when extracurricular engagement is also low.

Despite the restricted time, student perceptions of PE were generally positive. Many students viewed PE as equal or of greater importance than academic subjects, and over 30% expressed a desire for increased PE hours. This enthusiasm is consistent with previous studies, which indicate that when PE is delivered effectively, students value it for its physical benefits and contributions to enjoyment, social bonding, and emotional regulation (43). However, the current findings also point to a gap between students' attitudes and their actual participation in PA outside of school, with many reporting low levels of extracurricular engagement.

Interestingly, a perceptual gap emerged between students' high valuation of after-school PA and their low participation rates. While a majority of students agreed with the importance of extracurricular PA, 64.3% reported not participating in any such activities. This discrepancy suggests that students may appreciate the value of PA yet face barriers that prevent engagement, such as lack of time, limited access to facilities, transportation issues, or competing academic demands. This finding aligns with earlier research, which highlights that positive attitudes alone are insufficient predictors of behavior, particularly when structural or contextual limitations exist. Future interventions should aim not only to strengthen positive attitudes but also to remove practical barriers to participation, such as improving access to after-school sports programs, fostering community partnerships, or providing transportation support.

One notable divergence in this study relates to stakeholders' perceptions of environmental opportunities for PA. While most students reported a lack of accessible sports activities in their surroundings, PE teachers and administrators were more likely to rate these opportunities as moderate or high. This discrepancy may reflect differences in awareness, access, or expectations. Students' limited mobility or knowledge of available programs may contribute to underutilizing existing community resources. Alternatively, it may indicate a lack of structured opportunities in particular geographic or socioeconomically disadvantaged areas.

The analysis also highlighted the influence of PE exposure on student perceptions. Students receiving more than one hour of PE per week were significantly more likely to value PA and report better access to local sports opportunities. This aligns with previous research suggesting that greater exposure to PE can enhance students' motivation, self-efficacy, and positive attitudes toward an active lifestyle (44). Increasing PE hours may, therefore, serve as a viable strategy for improving fitness outcomes and instilling long-term habits and appreciation for PA.

Students generally viewed PE teachers as supportive and encouraging, especially regarding the importance of PA. However, follow-up engagement and integrating PA principles into daily life, such as promoting walking or using stairs, received more neutral responses. This indicates that while PE teachers fulfill a motivational role within class sessions, their influence may be limited beyond scheduled instruction. These findings echo previous literature emphasizing the need for professional development that equips PE teachers with tools to extend their impact into students' broader lifestyle behaviors (45).

Institutional barriers were also reported, with students noting that PE classes are canceled more frequently than other subjects and that encouragement for after-school activities remains weak. These findings suggest systemic de-prioritization of PE within school scheduling and policy implementation. Administrative commitment and structured school-wide strategies are essential for embedding PA into the broader educational culture (46). Even well-trained teachers may struggle to implement comprehensive and sustainable programs without the support of institutional buy-in.

Interestingly, while potential barriers such as lack of infrastructure, equipment, or teacher development were recognized by students, they were generally rated as having a “slight effect”. This indicates that when students enjoy PE, they overlook logistical shortcomings, or it reflects limited awareness of the structural challenges teachers face. Still, frequent class cancellations and some emotional barriers, such as embarrassment during PE, were more prominent, pointing to areas requiring policy and pedagogical attention.

Several countries facing similar challenges in PE have implemented effective strategies to improve access and quality. For example, Finland has emphasized the integration of PE into the national curriculum by mandating minimum weekly hours and linking PA to well-being and academic outcomes (47). Canada has addressed equity in PE through province-specific frameworks, community partnerships, and inclusive policies to engage diverse populations (48). These approaches highlight the importance of national policy alignment, adequate teacher training, and investment in facilities and extracurricular programming. Lessons from these international examples could guide improvements in PE delivery in Israel, especially in under-resourced or culturally diverse settings such as East Jerusalem.

This study underscores the complex interplay between institutional structures, stakeholder perceptions, and student behavior in school-based PA. While students express a strong appreciation for PE, limited instructional time, perceived environmental constraints, and inconsistent institutional support may impede broader engagement. A coordinated effort involving policy reform, professional development, and cross-sector collaboration is needed to create a more active and health-promoting educational environment in East Jerusalem schools. While this study provides valuable insights into stakeholder perceptions of PE within East Jerusalem schools, its geographic limitation may affect the generalizability of the findings. East Jerusalem represents a unique socio-political and cultural context within Israel, which may not fully reflect the conditions and attitudes present in other regions or educational sectors, such as secular Jewish, ultra-Orthodox, or mixed cities. To build on these findings and enhance their applicability, future research should aim to include a more diverse sample, encompassing schools from various geographic areas, socio-economic backgrounds, and demographic compositions across Israel. Such expansion would enable a more comprehensive understanding of national trends and facilitate the development of tailored, evidence-based policies to strengthen PE programs nationwide. Additionally, it is essential to recognize that the perspectives presented by different stakeholder groups may be influenced by response bias. For example, PE teachers may have a vested interest in portraying PE in a more favorable light, potentially overstating its institutional support or minimizing barriers. Similarly, students' responses may be influenced by personal enjoyment of PE or dissatisfaction with its current structure, while administrators may present responses aligned with institutional narratives or priorities. These potential biases should be considered when interpreting the findings, as they may influence the extent to which stakeholder perceptions accurately reflect the objective reality of PE implementation and support in schools. Future studies may benefit from triangulating stakeholder input with objective observations, curriculum analysis, or policy reviews to mitigate subjectivity.

In conclusion, educational policymakers and school administrators should consider increasing the allocated hours for PE, ensuring it is given equal priority alongside other academic subjects. Additionally, targeted investment in infrastructure, such as access to gymnasiums and open spaces, is essential to enhance the quality and consistency of PE delivery. Policymakers should also support the development of structured extracurricular PA programs, particularly in under-resourced schools, to provide students with meaningful opportunities beyond the classroom. Finally, establishing monitoring frameworks and stakeholder feedback mechanisms can help ensure that policy reforms are responsive, equitable, and sustainable across different school sectors in Israel.

## Data Availability

The original contributions presented in the study are included in the article/Supplementary Material, further inquiries can be directed to the corresponding authors.

## References

[1] Li Z Li J Kong J Li Z Wang R Jiang F. Adolescent mental health interventions: a narrative review of the positive effects of physical activity and implementation strategies. Front Psychol. (2024) 15:1433698. 10.3389/fpsyg.2024.1433698 38993342 PMC11236730

[2] Quiroz-Cárdenas F López-Gil JF. The role of school-based health centers in adolescent well-being: a call for action. Front Public Health. (2025) 13:1557124. 10.3389/fpubh.2025.1557124 40098800 PMC11911199

[3] Lyu S Zhang W. Opening the window to the children’s mind: the superior efficacy of open-ended physical games in the development of attention and socio-emotional skills. Front Psychol. (2025) 16:1511559. 10.3389/fpsyg.2025.1511559 40045973 PMC11879968

[4] Chen C Nibbio G Kotozaki Y. Editorial: Cognitive and mental health improvement under-and post-COVID-19. Front Psychol. (2025) 16:1565941. 10.3389/fpsyg.2025.1565941 40115284 PMC11924046

[5] Hashemzadeh Z Saberi H Aali SH. The study of the effectiveness of teaching philosophy in the form of a loop and exploring the working memory of blind students. Iran J Educ Sociol. (2022) 1(10):171–80. Available online at: https://qijes.com/index.php/ijes/article/view/799

[6] Wong KK Papachristou E Francesconi M Dekkers TJ. Editorial: “like a bee and a flower”—the symbiotic relationship between physical environment and children and young people’s psychosocial outcomes. Child Adolesc Ment Health. (2025) 30(2):115–8. 10.1111/camh.12773 40134337

[7] Li N Wang D Zhao X Li Z Zhang L. The association between physical exercise behavior and psychological resilience of teenagers: an examination of the chain mediating effect. Sci Rep. (2024) 14(1):9372. 10.1038/s41598-024-60038-1 38654069 PMC11039466

[8] Moghimi E Belfry K Farr S Stafford S Bogdan A Brush M Using a co-design approach to develop a preventative online mental health program for youth (POMHPY): a quality improvement project. BMC Health Serv Res. (2025) 25(1):219. 10.1186/s12913-024-12101-w 39920706 PMC11806559

[9] Pliogou V Tromara S Hajisoteriou C Angelides P. Preventing and combating school-related gender-based violence (SRGBV): laying the foundations for a safe, equitable, and inclusive school. Front. Educ. (2025) 10:1520731. 10.3389/feduc.2025.1520731

[10] Silva AFD Martins PC Santiago LN Silva DAS. Mapping evidence on integrated 24-hour movement behaviors in children and adolescents: a scoping review of reviews. Children (Basel). (2025) 12(3):260. 10.3390/children12030260 40150543 PMC11940917

[11] de Almeida AA Noll M. Physical activity and lifestyle behaviors in children and adolescents. Children (Basel). (2024) 11(11):1403. 10.3390/children11111403 39594978 PMC11592424

[12] Larsen MN Mohr M Lobelo F Frediani JK. Editorial: recreational team sports: prevention, treatment, and rehabilitation of non-communicable diseases. Front Public Health. (2025) 13:1549065. 10.3389/fpubh.2025.1549065 39885923 PMC11780590

[13] Uzunçıbuk H Fiorillo L Ronsivalle V Russo D Cicciù M Marrapodi MM Unraveling the complexities of sleep disturbances: a scientific perspective. J Contemp Dent Pract. (2024) 25(12):1081–3. 10.5005/jp-journals-10024-3784 40079984

[14] Hood AM Weiss KE Forsner M. Editorial: women in science: pediatric pain. Front Pain Res. (2025) 6:1539856. 10.3389/fpain.2025.1539856 PMC11961990 40176846

[15] Committee on Physical Activity and Physical Education in the School Environment, Food and Nutrition Board, Institute of Medicine, Kohl HW III Cook HD. Educating the Student Body: Taking Physical Activity and Physical Education to School. Washington (DC): National Academies Press (US) (2013). 3, Physical Activity and Physical Education: Relationship to Growth, Development, and Health. 24851299

[16] Hernández-González V Carné-Torrent JM Jové-Deltell C Reverter-Masia J. Global research trends on physical education practices: a bibliometric analysis and science-mapping study. Front Sports Act Living. (2025) 7:1532754. 10.3389/fspor.2025.1532754 40181928 PMC11965659

[17] Farooq MA Parkinson KN Adamson AJ Pearce MS Reilly JK Hughes AR Timing of the decline in physical activity in childhood and adolescence: Gateshead Millennium Cohort Study. Br J Sports Med. (2018) 52(15):1002–6. 10.1136/bjsports-2016-096933 28288966 PMC6204977

[18] Delfino LD Dos Santos Silva DA Tebar WR Zanuto EF Codogno JS Fernandes RA Screen time by different devices in adolescents: association with physical inactivity domains and eating habits. J Sports Med Phys Fitness. (2018) 58(3):318–25. 10.23736/S0022-4707.17.06980-8 28462567

[19] Delfino LD Tebar WR Silva DAS Gil FCS Mota J Christofaro DGD. Food advertisements on television and eating habits in adolescents: a school-based study. Rev Saude Publica. (2020) 54:55. 10.11606/s1518-8787.2020054001558 32491114 PMC7263799

[20] World Health Organization. Promoting Physical Activity Through Schools: A Toolkit. Geneva: World Health Organization (2021). Available online at: https://books.google.com/books?hl=en&id=CXdyEAAAQBAJ

[21] Harvey SP Markenson D Gibson CA. Assessing school wellness policies and identifying priorities for action: results of a bi-state evaluation. J Sch Health. (2018) 88(5):359–69. 10.1111/josh.12619 29609219

[22] UNESCO. Quality Physical Education (QPE) Guidelines for Policy-Makers. Paris: United Nations Educational, Scientific and Cultural Organization (2021). Available online at: https://unesdoc.unesco.org/ark:/48223/pf0000261795

[23] UNESCO. (2015). World-wide Survey of School Physical Education: Final Report. Paris: UNESCO. Available online at: https://unesdoc.unesco.org/ark:/48223/pf0000232993

[24] Shirotriya AK Beighle A. Physical education moving from marginalized to materialized. J Phys Educ Recreat Dance. (2023) 94(9):39–43. 10.1080/07303084.2023.2260440

[25] Tesler R Kolobov T Ng KW Shapiro E Walsh SD Shuval K Ethnic disparities in physical activity among adolescents in Israel. Am J Health Behav. (2019) 43(2):337–48. 10.5993/AJHB.43.2.10 30808473

[26] Alkaher I Goldman D Sagy G. Culturally based education for sustainability—insights from a pioneering ultraorthodox city in Israel. Sustainability. (2018) 10(10):3721. 10.3390/su10103721

[27] Zach S Dunsky A Stein H Litvin O Hellerstein D. Novice physical education teachers in Israel: facilitators and barriers to persistence in the profession. Sustainability. (2020) 12(9):3830. 10.3390/su12093830

[28] Ministry of Education (MOE). Guidelines for Physical Education Curriculum Implementation. Israel: Ministry of Education (2019).

[29] Carvalho GS Vilaça T. Editorial: health promotion in schools, universities, workplaces, and communities. Front Public Health. (2024) 12:1528206. 10.3389/fpubh.2024.1528206 39703484 PMC11655455

[30] Keikha M Ebrahimi MH Dianati-Nasab M Heidari-Beni M. Editorial: interventions to prevent or reduce unhealthy habits in children and adolescents during restricted conditions. Front Public Health. (2024) 12:1517367. 10.3389/fpubh.2024.1517367 39720802 PMC11667261

[31] Deng Y Li X Huang J Haegele JA Smith B Williams TL School-based factors influencing physical activity participation in children and adolescents with disabilities: a qualitative systematic review and meta-synthesis. Disabil Health J. (2025) 18(1):101707. 10.1016/j.dhjo.2024.101707 39322481

[32] Taylor IM Spray CM Pearson N. The influence of the physical education environment on children’s well-being and physical activity across the transition from primary to secondary school. J Sport Exerc Psychol. (2014) 36(6):574–83. 10.1123/jsep.2014-0038 25602140

[33] Richards KAR Pennington CG Sinelnikov OA. Teacher socialization in physical education: a scoping review of literature. Kinesiol Rev. (2019) 8(2):86–99. 10.1123/kr.2018-0003

[34] Kuhn AP Thompson HR Webster CA Burgeson C Chriqui J Okutoyi T Physical education Teachers’ perceived effectiveness in association with student attendance, teacher adaptability, external educational supports, and teaching format during the COVID-19 pandemic. J Healthy Eating Act Living. (2022) 2(3):97–112. PMC10521999 37771476

[35] World Health Organization. Guidelines on Physical Activity and Sedentary Behaviour. Geneva: WHO (2020). Available online at: https://www.who.int/publications/i/item/9789240015128 33369898

[36] Bull FC Al-Ansari SS Biddle S Borodulin K Buman MP Cardon G World Health Organization 2020 guidelines on physical activity and sedentary behaviour. Br J Sports Med. (2020) 54(24):1451–62. 10.1136/bjsports-2020-102955 33239350 PMC7719906

[37] OECD. Education at a Glance 2021: OECD Indicators. Paris: OECD Publishing (2021). 10.1787/b35a14e5-en

[38] Department of Education and Knowledge (ADEK). Physical Education and School Sports (ADEK School PE and School Sports Policy_v.1.1). Abu Dhabi: ADEK. (2025). Available online at: https://adek.gov.ae/-/media/Project/TAMM/ADEK/Policies/School-Policies/Health-safety-and-wellbeing/ADEK_S_PE-and-School-Sports-Policy_EN.pdf (Accessed April 12, 2025).

[39] GEMS Founders School Dubai. Physical activity policy 2023–2024. (2023). Available online at: https://www.gemsfoundersschool-dubai.com/en/-/media/project/gems/gfs_gems_founder_school_al_barsha/_files-and-documents/gfs-key-documents-and-guidelines/gfs-policy-13-october-2023/gfs-physical-activity-policy-2023-2024.pdf (Accessed July 20, 2025).

[40] Mesaieed International School. Physical education at MIS. Qatar Petroleum (2025). Available online at: https://www.mis.qp.qa/Pages/PE.aspx (Accessed April 12, 2025).

[41] Qatar News Agency. MOEHE affirms keenness to update physical education curriculum to develop students’ basic competencies. (2022). Available online at: https://www.qna.org.qa/en/News-Area/News/2022-02/07/0037-moehe-affirms-keenness-to-update-physical-education-curriculum-to-develop-students%27-basic-competencies (Accessed July 20, 2025).

[42] UNESCO. Quality physical education: Guidelines for policy-makers. United Nations Educational, Scientific and Cultural Organization (2015). Available online at: https://unesdoc.unesco.org/ark:/48223/pf0000231101 (Accessed July 26, 2025).

[43] Vega-Ramírez L Vidaci A Hederich-Martínez C. The effect of group work on expressive-artistic activities for the emotional regulation of university students. Educ Sci. (2022) 12(11):777. 10.3390/educsci12110777

[44] Zhang X Wang Y Leung SO. Technology acceptance model (TAM) and sports bracelets usage in physical education for freshmen: the role of gender and self-efficacy. Technol Pedagogy Educ. (2022) 32(1):45–63. 10.1080/1475939X.2022.2152861

[45] Castillo I Molina-García J Estevan I Queralt A Álvarez O. Transformational teaching in physical education and students' leisure-time physical activity: the mediating role of learning climate, passion and self-determined motivation. Int J Environ Res Public Health. (2020) 17(13):4844. 10.3390/ijerph17134844 32635673 PMC7370029

[46] Webster CA Glascoe G Moore C Dauenhauer B Egan CA Russ LB Recommendations for Administrators’ involvement in school-based health promotion: a scoping review. Int J Environ Res Public Health. (2020) 17(17):6249. 10.3390/ijerph17176249 32867355 PMC7503319

[47] Tammelin T Laine K Turpeinen S. Physical activity and school curriculum: Finnish experiences. WHO Regional Office for Europe (2016). Available online at: https://www.euro.who.int/__data/assets/pdf_file/0020/303482/Policy-Brief-3-Finland-final-edit-27-Oct-2016.pdf (Accessed July 20, 2025).

[48] ParticipACTION. The brain+body equation: Canadian kids need active bodies to build their best brains. The 2018 ParticipACTION report card on physical activity for children and youth. (2018). Available online at: https://www.chrome-extension://efaidnbmnnnibpcajpcglclefindmkaj/; https://www.participaction.com/wp-content/uploads/2022/09/2018_participaction_report_card_-_highlight_report_0.pdf (Accessed July 20, 2025).

